# Intracerebroventricular administration of leptin increase physical activity but has no effect on thermogenesis in cold-acclimated rats

**DOI:** 10.1038/srep11189

**Published:** 2015-06-08

**Authors:** Gang-Bin Tang, Xiang-Fang Tang, Kui Li, De-Hua Wang

**Affiliations:** 1 State Key Laboratory of Integrated Management of Pest Insects and Rodents, Institute of Zoology, Chinese Academy of Sciences, 1 Beichen West Road, Chaoyang, Beijing 100101, China; 2 State Key Laboratory of Animal Nutrition, Institute of Animal Sciences, Chinese Academy of Agricultural Sciences, 2 Yuanmingyuan West Road, Haidian, Beijing 100193, China

## Abstract

Most small homotherms display low leptin level in response to chronic cold exposure. Cold-induced hypoleptinemia was proved to induce hyperphagia. However, it is still not clear whether hypoleptinemia regulates energy expenditure in cold condition. We try to answer this question in chronic cold-acclimated rats. Results showed that 5-day intracerebroventricular(ICV) infusion of leptin (5 μg/day) had no effects on basal and adaptive thermogenesis and uncoupling protein 1 expression. Physical activity was increased by leptin treatment. We further determined whether ghrelin could reverse the increasing effect of leptin on physical activity. Coadministration of ghrelin (1.2 μg/day) completely reversed the effect of leptin on physical activity. Collectively, this study indicated the regulation of leptin on energy expenditure during cold acclimation may be mainly mediated by physical activity but not by thermogenesis. Our study outlined behavioral role of leptin during the adaptation to cold, which adds some new knowledge to promote our understanding of cold-induced metabolic adaptation.

Maintaining of the core body temperature in a cold environment is crucial of the survival of homeotherms. To maintain body temperature, homeotherms exhibit remarkable adaptations in physiology and behavior during the challenge of low environmental temperature^1^,^2^. These physiological and behavior adaptations generate a unique situation characterized by a chronic hypermetabolic state, in which increases of energy intake and thermogenesis are prerequisites of survival. Cold-exposed animals may thus represent interesting tools for investigations of the mechanisms underlying energy balance^3^.

Defense of core body temperature (Tc) can be energetically costly; thus it is critical that thermoregulatory circuits are modulated by signals of energy availability. Leptin, produced primarily in the adipose tissue, communicates the level of energy reserves in the periphery to the central nervous system (CNS)^4^,^5^,^6^,^7^,^8^,^9^. During cold acclimation, most small endothermic mammals decrease circulating leptin levels^10^,^11^,^12^,^13^,^14^,^15^. Several studies proved that hypoleptinemia in cold-acclimated small mammals serves as a starvation signal informing the energy deficient status to the brain, accordingly contributes to the subsequent hyperphagia^15^,^16^,^17^.

Leptin is also reported to promote energy expenditure in normal condition^18^,^19^. Mutations to the leptin or leptin receptor genes are associated with cold intolerance in ob/ob and db/db mice^20^,^21^,^22^, suggesting that the intact leptin signaling pathway seems to be a prerequisite for the survival of small endothermic mammals in cold. However, ob/ob mice can survive in extreme cold at 4 °C, provided they are adapted to the cold by gradually lowering ambient temperature^23^. Another study showed that even deletion of leptin receptor in 75% of hypothalamic neurons could not change the ability of cold tolerance^24^. On the basis of these studies, it is highly uncertain whether leptin involves in the regulation of energy expenditure during cold adaptation, especially in non-mutant models.

Ghrelin, a stomach-derived hormone also plays a role in long-term regulation of energy balance^25^. In normal condition, leptin and ghrelin were proposed to operate as functional antagonists in the control of metabolism and energy homeostasis^26^. In cold condition, our previous study reported that leptin treatment decreased serum ghrelin levels, suggesting an interaction between them in cold. However, ghrelin was not involved in the regulation of the effect of leptin on food intake and body weight^27^, raising the possibility that these two hormones interact to direct an appropriate regulation on energy expenditure.

In this study, we used chronic cold-acclimated rats as model and aimed to answer the following questions: 1) Does leptin regulate energy expenditure through modulating physical activity? 2) Does leptin regulate energy expenditure through modulating thermogenesis? 3) Is ghrelin involved in the regulation of the effect of leptin on energy expenditure?

## Results

### Experiment 1 Intracerebroventricular (ICV) infusion of leptin increase physical activity but has no effect on metabolism in cold-acclimated rats

No significant effects of leptin treatment were found on resting metabolic rate (RMR) and non-shivering thermogenesis (NST) (Table 1). Body temperature remained constant during the treatment (Table 1). In addition, there was also no significant change in uncoupling protein 1 (UCP1) protein levels (Table 1; Supplementary Figure S1).

**Figure 1:**
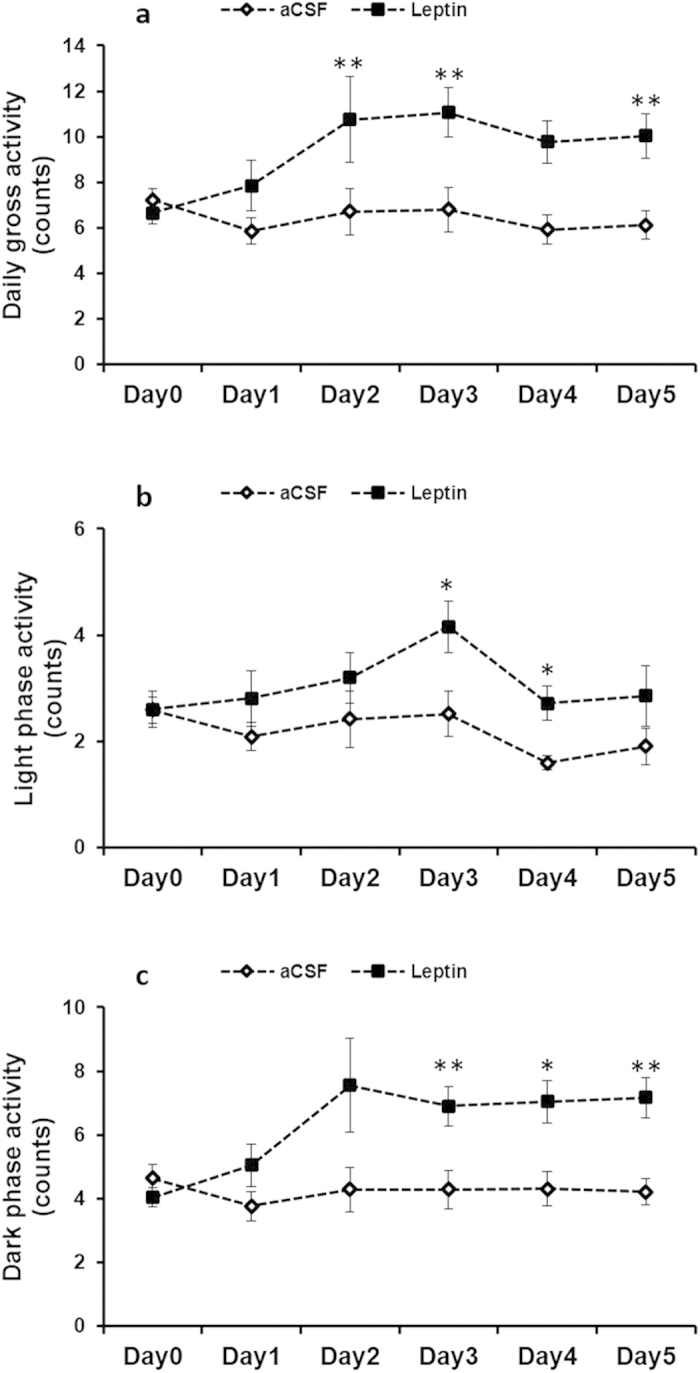
Daily physical activity (**a**), light phase activity (**b**) and dark phase activity (**c**) in control and leptin-treated cold-acclimated rats. “*”, p < 0.05, “**”, p < 0.01. All error bars show s.e.m.

Chronic leptin treatment resulted in marked increase in physical activity (Group effect, F_1,16_ = 6.485, P = 0.022; day effect, F_5,80_ = 5.430, P < 0.001; interaction group × day, F_5,80_ = 5.822, P < 0.001; Fig. 1a). Compared to control group, average physical activity in leptin group was increased by 33% during 5-day treatment. At the end of the experiment, ICV administration of leptin induced significant increases in both light phase (Group effect, F_1,16_ = 6.268, P = 0.024; day effect, F_5,80_ = 2.644, P = 0.029; interaction group × day, F_5,80_ = 1.112, P = 0.361; Fig. 1b) and dark phase activity (Group effect, F_1,16_ = 6.485, P = 0.022; day effect, F_5,80_ = 5.430, P < 0.001; interaction group × day, F_5,80_ = 5.822, P < 0.001; Fig. 1c). Average physical activity in light phase and in dark phase were increased by 55% and 60% respectively, compare to the control groups.

Leptin administration induced significant decreases of serum ghrelin levels (T_16_ = 3.923, P = 0.01; Supplementary Table S1). Compared to control group, serum ghrelin level was decreased by 49% in leptin group. Serum triiodothyronine, norepinephrine and cathecolamine levels were not affected by leptin treatment (Supplementary Table S1).

### Experiment 2 Ghrelin administration reversed the effect of leptin on physical activity in cold-acclimated rats

**Figure 2:**
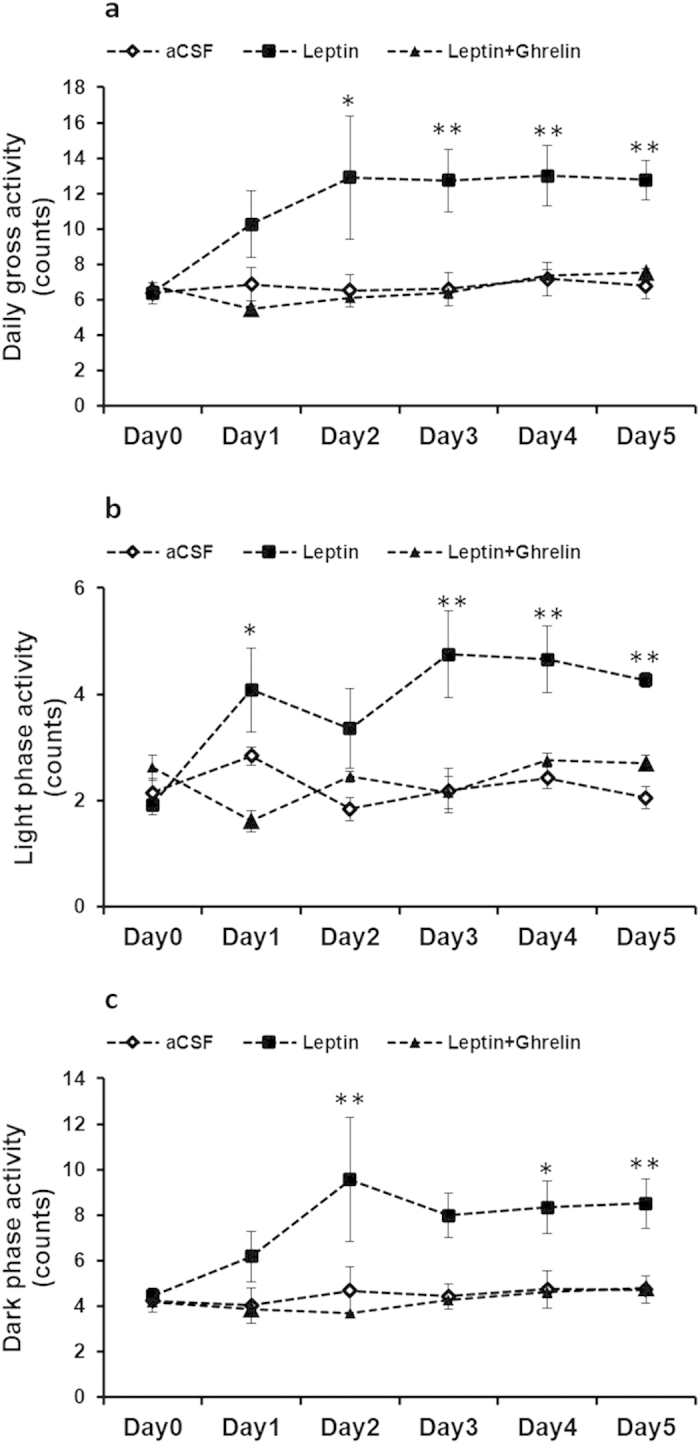
Daily physical activity (**a**), light phase activity (**b**) and dark phase activity (**c**) in control, leptin, leptin plus ghrelin treated cold-acclimated rats. “*”, p < 0.05, “**”, p < 0.01. All error bars show s.e.m.

Significant differences in daily physical activity (Group effect, F_2,14_ = 6.29, P = 0.011; day effect, F_5,70_ = 4.648, P = 0.001; interaction group × day, F_10,70_ = 3.547, P = 0.001; Fig. 2a), light phase activity (Group effect, F_2,14_ = 9.485, P = 0.002; day effect, F_5,70_ = 3.657, P = 0.005; interaction group × day, F_10,70_ = 4.347, P < 0.001; Fig. 2b) and dark phase activity (Group effect, F_2,14_ = 5.027, P = 0.023; day effect, F_5,70_ = 4.328, P = 0.002; interaction group × day, F_10,70_ = 2.821, P = 0.005; Fig. 2c) of three groups were detected during the 5 days’ treatment. The activities in leptin plus ghrelin group was similar to control group. Therefore, the increasing effects of leptin on physical activities were completely reversed by 5 day’s ghrelin treatment.

## Discussion

In normal condition, leptin plays a pivotal role in promotion of thermogenesis^18^,^19^. In cold condition, if leptin acts in the same way, then cold-induced hypoleptinemia would inhibit thermogenesis. This seemingly paradoxical question was previously discussed but was never resolved^15^,^17^,^28^. In this study, we present evidence that central leptin administration did not affect basal thermogenesis, non-shivering thermogenesis and core body temperature. In support of this, serum norepinephrine and cathecolamine concentrations, two key hormones involving in the regulation of adaptive thermogenesis, were not affected by leptin administration. The hypothalamic-pituitary-thyroid (HPT) axis represents the major endocrine system that participates in the regulation of energy expenditure^29^,^30^. Unchanged serum T3 levels in the present study indicated leptin administration had no effect on HPT axis in chronic cold-acclimated rats. In addition, interscapular brown adipose tissue (iBAT) UCP1 protein levels were also not affected by leptin treatment. Taken together, the present results lend support for the notion that leptin may not play a key role in thermogenesis in chronic cold-acclimated rats.

Physical activity is positively associated with energy expenditure^31^,^32^. Leptin-deficient ob/ob mice have profoundly decreased locomotor activity^8^. Treatment of ob/ob mice for three weeks with exogenous leptin increased locomotor activity and substantially decreased adiposity^8^. In wild type rats, ICV leptin treatment resulted in a significant increase in spontaneous physical activity (SPA) in room temperature^33^. The role of leptin in the regulation of activity has become an increasing focus of research because increased activity is a key determinant in negative energy balance induced by leptin, which cannot be accounted for solely by the leptin-induced food intake reduction^34^.

It is a big challenge for small homotherms to maintain core body temperature in response to chronic cold exposure, since thermogenesis is energetically costly. Aside from increased food intake, they also need to allocate energy which is used for activity, growth and reproduction to thermogenesis. In the present study, we found that exogenous leptin caused increased physical activity in cold-exposed rats in spite of the fact that they suffered energy deficiency. This finding suggests that physical activity is under the strict regulation of leptin in cold condition. Cold-induced hypoleptinemia can be speculated as a regulator, which allows for hypoactivity. It is reasonable that cold-exposed animals would save lots of energy from this behavioral adjustment.

Interaction between leptin and ghrelin is proposed to be important for energy balance^35^. In this study, 5-day leptin treatment in cold-exposed rats caused a reduction of ghrelin. This was consistent with the previous study^27^. In chronic cold-exposed rats, ghrelin may not be involved in the regulation of energy intake and thermogenesis, since ghrelin administration did not change the food intake and thermogenesis compared to the leptin-treated animals^27^. Chronic peripheral or ICV infusions of ghrelin were reported to reduce spontaneous activity^36^,^37^. Our and other findings make it possible that ghrelin may regulate energy balance by modulating spontaneous activity. Actually, we found that ghrelin administration completely reversed the increasing effect of leptin on physical activity, suggesting that the regulatory effect of leptin on physical activity at least in part be mediated by ghrelin.

Our and other studies uncovered a complex but precise regulation during cold-induced metabolic adaptation, in which leptin behaves both physiologically and behaviorally. On one hand, hypoleptinemia acts as a starvation signal to increase food intake. On the other, it suppresses locomotors activity to reduce unnecessary energy expenditure, which was involved in ghrelin. Expanding the physiological adjustment of energy balance to include mechanisms regulating physical activity may promote our understanding of cold-induced metabolic adaptation.

In summary, we presented evidence that chronic ICV administration of leptin had no effects on basal and adaptive thermogenesis, while it significantly increased physical activity in cold-acclimated rats. We also found that the effect of leptin on physical activity is mainly mediated by ghrelin.

## Methods

### Ethics Statement

Animal care and experiments were conducted in accordance with the protocols approved by Animal Experimentation Ethical Committee of Institute of Zoology, the Chinese Academy of Sciences following the Animal Care and Use Committee guideline.

### Rats

Adult male Wistar rats were maintained at 23 ± 1 °C with a 12 L:12D photoperiod (lights on at 08:00). Food and water were provided ad libitum. All animal procedures were approved by the Animal Care and Use Committee of Institute of Zoology, the Chinese Academy of Sciences.

### Peptides

Leptin was obtained from R&D Systems Inc. (Minneapolis, MN, USA). Biologically active rat ghrelin was purchased from Phoenix Pharm. Inc. (Burlingame, CA, USA).

### ICV infusion

Animals were anesthetized with intraperitoneal (i.p.) injection of sodium pentobarbital (60 mg/kg). 27-gauge stainless-steel cannulae (Alza Corp. Palo Alto, CA, USA) were stereotaxically implanted aimed at the lateral ventricle (stereotaxic coordinates from Bregma: 0.8 mm caudal, 1.6 mm lateral, 3.2 mm ventral). Following surgery, the rats were allowed to recover for 6 days. Angiotensin II drinking tests were performed at the end of the recovery period. Rats that drank ≥5 ml of water in 30 min after the ICV injection of 100 ng/10ul angiotensin II (Phoenix Pharm. Inc, Burlingame, CA, USA) were considered to have the cannula correctly placed for ICV microinjection. On the test day, the animals were anesthetized with isoflurane, and each was surgically implanted with an Alzet osmotic minipump 2001 (Alza Corp. Palo Alto, CA, USA) filled with artificial cerebrospinal fluid (aCSF) or hormones by insertion under the skin over the the dorsal side. A catheter from the minipump was connected into the ICV cannula through a subcutaneous tunnel.

## Experimental protocols

### Experiment 1: Effect of chronic administration of leptin on physical activity, basal and adaptive thermogenesis in cold-acclimated rats

In order to test the predication that leptin has a long-term role in the regulation of energy expenditure, we treated 18 cold-acclimated rats with either aCSF or leptin for 6 days: Control group (9 animals, maintained at 5 °C for 15 days and then received with ICV infusion of aCSF for another 5 days); Leptin group (9 animals maintained at 5 °C for 15 days and then received with ICV infusion of leptin for another 5 days). physical activity, RMR and NST and were measured during leptin administration.

### Experiment 2: Effect of coadministration of ghrelin on the regulation of physical activity in cold-acclimated rats

We examined the effect of leptin on physical activity and interaction between leptin and ghrelin during cold acclimation in this experiment. Rats were cold-acclimated (5 °C) for 15 days, then they were divided randomly into three groups: Control group (6 animals, free acess to food, given ICV infusion of aCSF for 5 days); Leptin group (6 animals, free acess to food, given ICV infusion of leptin for 5 days) (dosage:5 μg/day); Leptin plus ghrelin group (6 animals, free acess to food, given ICV infusion of leptin and ghrelin for 5 days) (Leptin: 5 μg/day; Ghrelin: 1.2 μg/day). Physical activities were measured.

### Core body temperature and physical activity

Core body temperature and physical activity were recorded telemetrically from the transmitter implanted in the abdomen (Mini Mitter, Model G2 E-Mitter, to ±0.1 °C in the temperature range of 33–41 °C). Individual cages were placed on the receiver board (Mini Mitter, Model ER-4000). All receivers and the DP-24 DataPort were connected to a computer with the VitalView software. Core body temperature and physical activity were recorded at 30-sec intervals throughout the experiment.

### Metabolic trials

Measurement and analysis of thermogenesis and thermoregulation were conducted as previously described^15^,^31^,^38^. Specifically, RMR was assessed from the rate of O_2_ consumption and CO_2_ production at 30 °C (constant-temperature incubator; Yiheng Corp., Shanghai, China) by using a LabMaster system (TSE-Systems, Bad Homburg, Germany)^31^. Individual animal was placed in a rat cage (15 L) for 2·h. The flow rate of air was 4000 ml · min^–1^. In each trial, up to three rats and one obligate reference channel were recorded in parallel, yielding a 3–6 min resolution of metabolic readings. The whole experimental procedure as well as data collection is automatically controlled by the software. RMR was estimated from the stable lowest rate of oxygen consumption over 6–12 min.

For the measurement of NST, single rat was received a subcutaneous injection of norepinephrine (0.4 mg kg^−1^)^31^,^39^. Oxygen consumption and carbon dioxide production were measured at 27 °C. NST was calculated from the highest rate of oxygen consumption over 6 min.

### Hormone assay

Leptin, ghrelin, norepinephrine, cathecolamine and triiodothyronine concentrations were measured by using ELISA kits (Leptin: Linco Research, St. Charles, MO; Ghrelin: Ever Systems Biology Laboratory,Inc. Sacramento, CA, USA; NE, CA, T3: Qi-Song Corp, Beijing, China).

### Measurement of UCP1 content in iBAT

Mitochondrial protein concentrations of BAT were determined by Folin phenol method with bovine serum albumin as standard^40^. iBAT UCP1 content was measured by Western blotting as described previously^15^. Specifically, total iBAT mitochondrial protein (50 μg per lane) was separated in a discontinuous SDS-polyacylamide gel (12.5% running gel and 3% stacking gel) and blotted to a nitrocellulose membrane (Hybond-C, Amersham, UK). After transfer, membrane were blocked in 5% milk in Tris-buffered saline-Tween for 1 h at room temperature and probed with the indicated antibodies overnight at 4 °C. Following incubation with the secondary antibody for 1 h, the bands were visualized by chemiluminescence (Amersham Life Sciences, Little Chalfont, UK). Quantification of the blots was determined with the use of a Quantity One Ver.4.4.0 (BioRad, USA). Primary antibodies used were as follows: rabbit anti-UCP1 (ab10983, Abcam, Cambridge, MA, USA), diluted 1:10,000; mouse anti-β-tubulin (E7, DSHB, Iowa City, Iowa, USA), diluted 1:5000. The secondary antibodies of goat anti-rabbit IgG (1:5,000; ZSGB-BIO Co., Beijing, CHN) and goat anti-mouse IgG (1:5,000; ZSGB-BIO Co., Beijing, CHN) were used.

### Statistical analysis

Data were analyzed using SPSS 13.0 software (SPSS Inc., Chicago, IL, USA). Prior to all statistical analyses, data were examined for normality of variance using the Kolmogorov-Smirnov test. Group differences in physical activity, and body temperature were assessed by two-tailed repeated measures ANOVA. Differences in metabolic rate and hormones was analyzed by two-tailed T test. Data are expressed as mean ± SEM, P < 0.05 was considered to be statistically significant.

## Additional Information

**How to cite this article**: Tang, G.-B. *et al.* Intracerebroventricular administration of leptin increase physical activity but has no effect on thermogenesis in cold-acclimated rats. *Sci. Rep.* **5**, 11189; doi: 10.1038/srep11189 (2015).

## Supplementary Material

Supplementary Information

## Figures and Tables

**Table 1:** **Metabolic profiles in control and leptin group**.

**Metabolic parameters**	**aCSF**	**Leptin**	**Difference**
RMR (ml O_2_.g^−1^.h^−1^)	1.26 ± 0.10	1.41 ± 0.09	No
NST (ml O_2_.g^−1^.h^−1^)	2.53 ± 0.03	2.25 ± 0.11	No
CBT (°C)	37.8 ± 0.18	37.9 ± 0.12	No
UCP1 (RU)	1.24 ± 0.17	1.39 ± 0.09	No

RMR, resting metabolic rate; NST, non-shivering thermogenesis; CBT, core body temperature; UCP1, uncoupling protein 1.

Data are expressed as mean ± SEM, P < 0.05 was considered to be statistically significant.
