# Supplementary material for: Intracerebroventricular administration of leptin increase physical activity but has no effect on thermogenesis in cold-acclimated rats
Source: Sci Rep. 2015 Jun 8;5:11189. doi: 10.1038/srep11189 (PMC4459185; doi:10.1038/srep11189)
Supplement: Supplementary Information [file srep11189-s1.pdf]

Intracerebroventricular administration of leptin increase physical activity but has no effect on thermogenesis in cold-acclimated rats

Gang-Bin Tang [a,*], Xiang-Fang Tang [b], Kui Li [b], De-Hua Wang [a,*]

[a] State Key Laboratory of Integrated Management of Pest Insects and Rodents, Institute of Zoology, Chinese Academy of Sciences, 1 Beichen West Road, Chaoyang，Beijing 100101, China

[b] State Key Laboratory of Animal Nutrition, Institute of Animal Sciences, Chinese Academy of Agricultural Sciences, 2 Yuanmingyuan West Road, Haidian, Beijing 100193, China

*** Corresponding author:**

Dr. Gang-bin Tang

Email: gbtang@126.com

Prof De-hua Wang

Email:wangdh@ioz.ac.cn

Supplementary files

Supplementary Figure S1 UCP1 expression in aCSF and leptin group

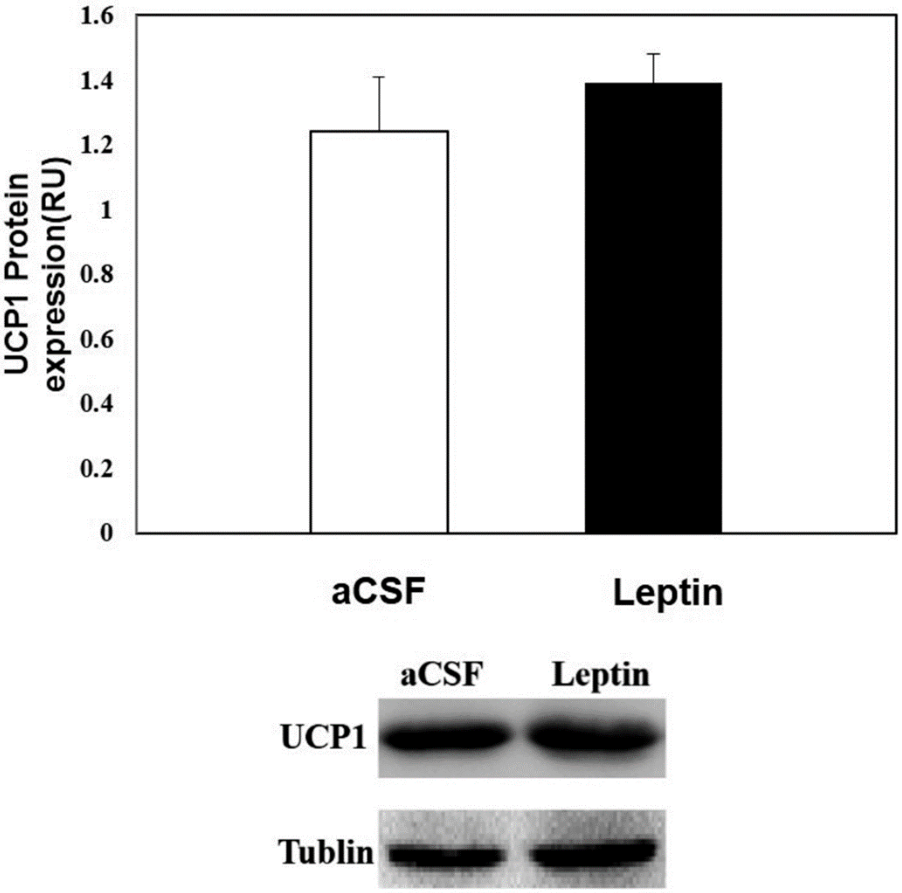

Supplementary Table S1 Serum hormones in control and leptin group.

Hormones	aCSF	Leptin	Difference
Leptin (ng/ml)	4.2±0.1	4.0±0.1	No
Ghrelin (pg/ml)	80.34±7.2	40.9±6.9	Yes
Triiodothyronine (ng/ml)	1.6±0.1	1.4±0.1	No
Norepinephrine (ng/ml)	337.8±5.5	337.2±9.4	No
Catecholamine (ng/ml)	532.5±6.9	532.6±6.9	No

Data are expressed as mean ±SEM, $P<0.05$ was considered to be statistically significant.
